# Distribution of the Sex-Determining Gene *MID* and Molecular Correspondence of Mating Types within the Isogamous Genus *Gonium* (Volvocales, Chlorophyta)

**DOI:** 10.1371/journal.pone.0064385

**Published:** 2013-05-16

**Authors:** Takashi Hamaji, Patrick J. Ferris, Ichiro Nishii, Yoshiki Nishimura, Hisayoshi Nozaki

**Affiliations:** 1 Department of Botany, Graduate School of Science, Kyoto University, Sakyo-ku, Kyoto, Japan; 2 Department of Ecology and Evolutionary Biology, University of Arizona, Tucson, Arizona, United States of America; 3 Temasek Life Sciences Laboratory, The National University of Singapore, Singapore, Singapore; 4 Department of Biological Sciences, Graduate School of Science, University of Tokyo, Bunkyo-ku, Tokyo, Japan; University of Connecticut, United States of America

## Abstract

**Background:**

Isogamous organisms lack obvious cytological differences in the gametes of the two complementary mating types. Consequently, it is difficult to ascertain which of the two mating types are homologous when comparing related but sexual isolated strains or species. The colonial volvocalean algal genus *Gonium* consists of such isogamous organisms with heterothallic mating types designated arbitrarily as *plus* or *minus* in addition to homothallic strains. Homologous molecular markers among lineages may provide an “objective” framework to assign heterothallic mating types.

**Methodology/Principal Findings:**

Using degenerate primers designed based on previously reported *MID* orthologs, the “master regulator” of mating types/sexes in the colonial Volvocales, *MID* homologs were identified and their presence/absence was examined in nine strains of four species of *Gonium*. Only one of the two complementary mating types in each of the four heterothallic species has a *MID* homolog. In addition to heterothallic strains, a homothallic strain of *G*. *multicoccum* has *MID*. Molecular evolutionary analysis suggests that *MID* of this homothallic strain retains functional constraint comparable to that of the heterothallic strains.

**Conclusion/Significance:**

We coordinated mating genotypes based on presence or absence of a *MID* homolog, respectively, in heterothallic species. This scheme should be applicable to heterothallic species of other isogamous colonial Volvocales including *Pandorina* and *Yamagishiella*. Homothallism emerged polyphyletically in the colonial Volvocales, although its mechanism remains unknown. Our identification of a *MID* homolog for a homothallic strain of *G*. *multicoccum* suggests a *MID*-dependent mechanism is involved in the sexual developmental program of this homothallic species.

## Introduction

Isogamy is a mode of sexual reproduction involving the agglutination and fusion of two gametes that are essentially identical in size and shape. Isogamous organisms are widespread in eukaryotes such as yeasts and algae. The genus *Gonium* comprises colonial volvocalean green algae consisting of 8-, 16- or 32-cells in the form of a curved plate; the isogametes of *plus* and *minus* of most *Gonium* species form tubular mating structures (TMS) at the base of the two flagella [1]. Nozaki [2] called this mode of TMS formation “bilateral mating papilla.” *G*. *multicoccum* gametes do not have any TMS [3].

*Chlamydomonas reinhardtii*, an isogamous single-celled green alga, has two genetically determined, heterothallic mating types: *plus* and *minus* [4]. It has been used to study molecular and cellular mechanisms of sexual development for over half a century. Although the gametic cell sizes of both mating types are similar, a *plus* gamete has a TMS or “fertilization tubule” filled with actin filaments, while a *minus* does not [5]–[8]. Such a mode of TMS formation is called “unilateral mating papilla” [2]. Recently, Mogi et al [9] immunostained actin localized to the TMS of activated gametes from both mating types in *G*. *pectorale*, suggesting a common subcellular architecture among the TMS of the unilateral and bilateral mating papilla.

Unilateral mating papilla may enable cytological determination of corresponding mating types across species, while bilateral mating papillae do not because they do not show any cytological gametic difference between the two. In the colonial Volvocales, more than one sexually isolated group or syngen is recognized in various morphological or taxonomic species (e.g. *Pandorina morum* [10]; *Gonium viridistellatum* [11]); correspondence based on crossing experiment is not definable even within a single species with bilateral mating papillae. Currently reported “mating types” of *Gonium* strains have been determined based on crossing examinations within species, although their designations as “*plus*” or “*minus*” are arbitrary and do not necessarily correspond to those of the other species. To solve this lack of conformity, an objective and easily accessible molecular marker should be established.

Such a marker should correspond to a conserved domain among lineages and cosegregate with one of the mating types. The *C*. *reinhardtii* mating type determining protein, minus dominance (MID), dominantly determines mating type *minus* as a transcription factor with a conserved putative DNA-binding RWP-RK domain [12]–[14], which served as a candidate sequence for designing degenerate primers for identification of homologs in colonial volvocalean algae, including two *Gonium* species [15]–[18]. *MID* homologs in reported organisms cosegregate with mating types or sexes, suggesting a conserved mechanism in sex determination/differentiation. Thus, *MID* is an outstanding candidate for a molecular correspondence of mating types over species with bilateral mating papilla.

Here we propose a novel set of objective mating types in the genus *Gonium*, based on molecular identification of *MID* homologs. Nine strains of four *Gonium* species were examined. Quite interestingly, not only heterothallic strains but also a homothallic strain (*G*. *multicoccum* NIES-1708 [19]) retain a *MID* homolog.

## Materials and Methods

### Strains and culture conditions

Strains were obtained from the Microbial Culture Collection at the National Institute of Environmental Studies (NIES) [20] as summarized in Table 1. Culture conditions were essentially the same as described previously [16].

**Table 1:** List of *Gonium* strains mentioned here.

Species	Strain	Mating type designation	*MID* Acc. No.	*MID*/*non-MID mating type* (if heterothallic)
*G*. *maiaprilis*	NIES-2455 (Asa041901, sampled 2004)	*plus*	–	*non-MID mating type*
*G*. *maiaprilis*	NIES-2457 (Asa041903, sampled 2004)	*minus*	AB623044 [18]	*MID mating type*
*G*. *multicoccum*	NIES-1038 (GQ-M-Tx-1 a)	No designation^b^	AB774225 (this study)	*MID mating type*
*G*. *multicoccum*	NIES-1039 (GQ-M-Tx-2 a)	No designation^b^	–	*non-MID mating type*
*G*. *multicoccum*	NIES-1708 (Asa.Goni.84, sampled 2004)	(homothallic)	AB774226 (this study)	(homothallic)
*G*. *octonarium*	NIES-851 (GO-LC-1+ a)	*plus*	–	*non-MID mating type*
*G*. *octonarium*	NIES-852 (GO-LC-3– a)	*minus*	AB774227 (this study)	*MID mating type*
*G*. *pectorale*	NIES-1710 (Kaneko3, sampled 2000)	*minus*	AB353340 [16]	*MID mating type*
*G*. *pectorale*	NIES-1711 (Kaneko4, sampled 2000)	*plus*	–	*non-MID mating type*
*G*. *quadratum*	NIES-652 (90-423-3, sampled 1989)	*minus*	AB774228 (this study)	*MID mating type*
*G*. *quadratum*	NIES-653 (90-423-2, sampled 1989)	*plus*	–	*non-MID mating type*
*G*. *viridistellatum*	NIES-654 (KY-4 (+), UTEX 2519, sampled 1980)	*plus*	AB774224 (this study)	*MID mating type*
*G*. *viridistellatum*	NIES-655 (KY-7 (–), UTEX 2520, sampled 1980)	*minus*	–	*non-MID mating type*

a The four strains were kindly provided by Dr. Richard C. Starr (The Culture Collection of Algae at the University of Texas at Austin) in 1994.

b These two strains are heterothallic and formed zygotes when mixed (Nozaki, unpublished data).

### Identification of *MID* homologs

Nested PCR with degenerate primers amplified partial regions of *MID* genes, based on which sequence-specific primers were designed for inverse PCR [21] or thermal asymmetric interlaced (TAIL) PCR [22] to sequence flanking regions (details are summarized in Text S1; primers are listed in Table 2).

**Table 2:** Primers used in this study.

Primer name	Sequence	Reference
dMT-dF3	RCIMRIAARGCIGAYYTIAC	[16]
mt-R4	ACYTTICKRWAIGGCCAICK	[15]
CCGMID-F1	AGGACTGCATGGACGCCTT	
Goni-MID-F	GAGTGGCTNAARGANTGCATGGA	
Goni-MID-R	ACCTTWCGRTANGGCCAANCG	
GmMIDbF	AAGAGCCTTGGCATCTCAACA	
GmMIDaR	TGGAAGTAAGAACTGATATCTGC	
GmHetBF	CCTCTGTCGTCAATTAGGCA	
GmHetAR	GCGCGGATATCAGCAACTAC	
GoMID-BF	ACGAATATGTCGTCAACTGG	
GoMID-AR	TCAAGTACGTTGTCGAAATC	
GoMID_Rev4	CACAGCAGAGCTCGAAGAACGT	
GoMID_Rev5	GCGTGATAATTGTTGCCTTGG	
GoMID_Rev6	AAGAGTCTGGTTCGCAATTACT	
GoMID_B_Fwd1	ATGGACGCTTTCAAGAAGCAGAT	
GoMID_B_Fwd2	CTGGACGGATACCGTCTTGATA	
GoMID_B_Fwd3	TTAGTACATCACGCAGAGCGGC	
GQM-F1	AATCCTGTCAGAGGGCTATCGGCTTGAA	
GQM-F2	TGCGTACCGCTTTCCCATGCGTTACATT	
GQM-F3	GACATCAGCGGCTACTTCCACTTACCTA	
GQM-R3	TAGGTAAGTGGAAGTAGCCGCTGATGTC	
GQM-R2	AATGTAACGCATGGGAAAGCGGTACGCA	
GQM-R1	TTCAAGCCGATAGCCCTCTGACAGGATT	
ITS_a_short	GTTTCCGTAGGTGAACCTGC	modified (BamHI site is omitted) from [23]
ITS_b_short	ATATGCTTAAGTTCAGCGGGT	modified (BamHI site is omitted) from [23]
Gmul-Fwd	GTGCATGGACGCCTTCCTTAAACA	
Gmul-Rev	TATGGCCAGCGTGGTATGCCTAAT	
Goct-Fwd	TGCATGGACGCTTTCAAGAAGCAG	
Goct-Rev	GTTGCCGGCATATGCGTTTCAGAT	
Gqua-Fwd	TCAAGCAAATCCTGTCAGAGGGCT	
Gqua-Rev	GAATGCCCAATTGGCGGCAAATAC	
Gvir-Fwd	ACTGCATGGACGCTTTCCTTCAAC	
Gvir-Rev	GCGGGATACCCAGTTGACGACATATT	

### Phylogenetic and molecular evolutionary analyses

Phylogenetic analyses were performed using two data sets. One consists of ClustalX 2.0 [24] -aligned entire protein sequences of eleven MID homologs of the Volvocales (Fig. S1). The other alignment is composed of amino acid sequences (47 aa, Fig. S2) of RWP-RK domains (the 25 RWP-RK containing proteins recognized in *C*. *reinhardtii* and *Volvox carteri* genome databases, http://www.phytozome.net/ Phytozome v8.0, Joint Genome Institute, Walnut Creek, CA, USA [25], [26], and the eleven *MID* homologs). Maximum likelihood (ML) method, based on Whelan and Goldman model (WAG) by PhyML 3.0 [27], [28], and ML and neighbor joining method, using Jones-Taylor-Thornton model by *MEGA* version 5, were carried out with bootstrap values from 1000 replications [29]–[32].

A molecular evolutionary analysis of non-synonymous and synonymous substitutions was performed by YN00 in the PAML package [33], [34].

## Results and Discussion

**Figure 1:**
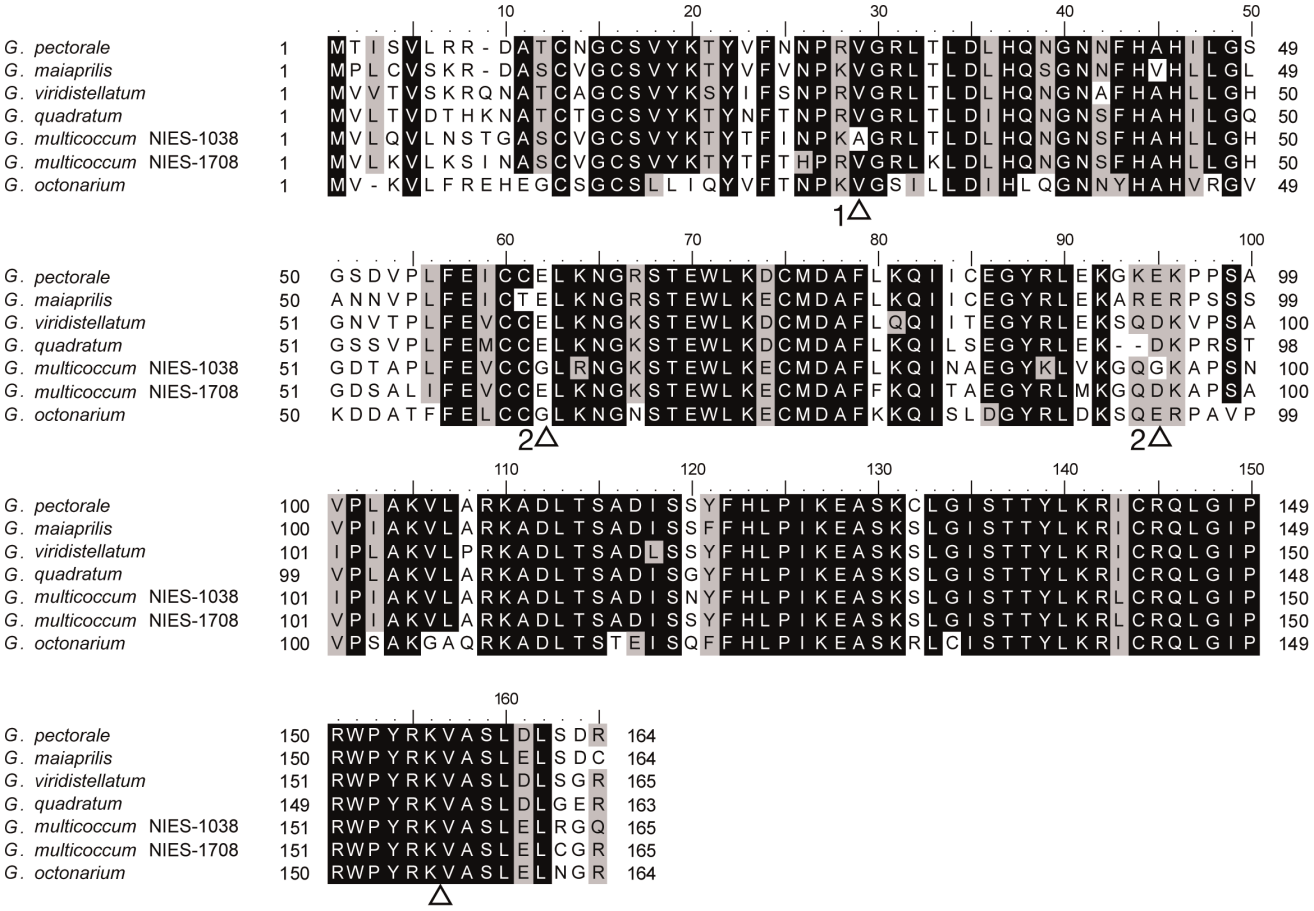
Alignment of seven MID homologs from *Gonium pectorale* NIES-1710, *G.maiaprilis* NIES-2457, *G*. *viridistellatum* NIES-654, *G*. *quadratum* NIES-652, *G*. *multicoccum* NIES-1038 (heterothallic), NIES-1708 (homothallic), and *G*. *octonarium* NIES-852. Solid and shaded backgrounds indicate identity or similarity over 80% of the sequences aligned, respectively. Triangles indicate intron sites and the numbers the positions in the codons unless between codons.

In our degenerate PCR-based approach, the *MID* homolog from every species of *Gonium* was obtained (Table 1). The primary data were genomic sequences, so the exon-intron structures of *MID* homologs were manually predicted based on *MID* genes of *G*. *pectorale* and *G*. *maiaprilis* (Figure 1 [16], [18]). The intron sites are exactly the same among the *Gonium* species; there are several insertion/deletion sites in the CDS. *MID* orthologs of *Gonium* have moderate%GC which is a common feature of *MID* (intron length and%GC summarized in Table S1). The putative DNA-binding RWP-RK domain-containing C terminus region of *MID* is well conserved within the genus *Gonium*, while the N terminus region is relatively more varied, consistent with earlier *MID* gene comparisons [13].

**Figure 2:**
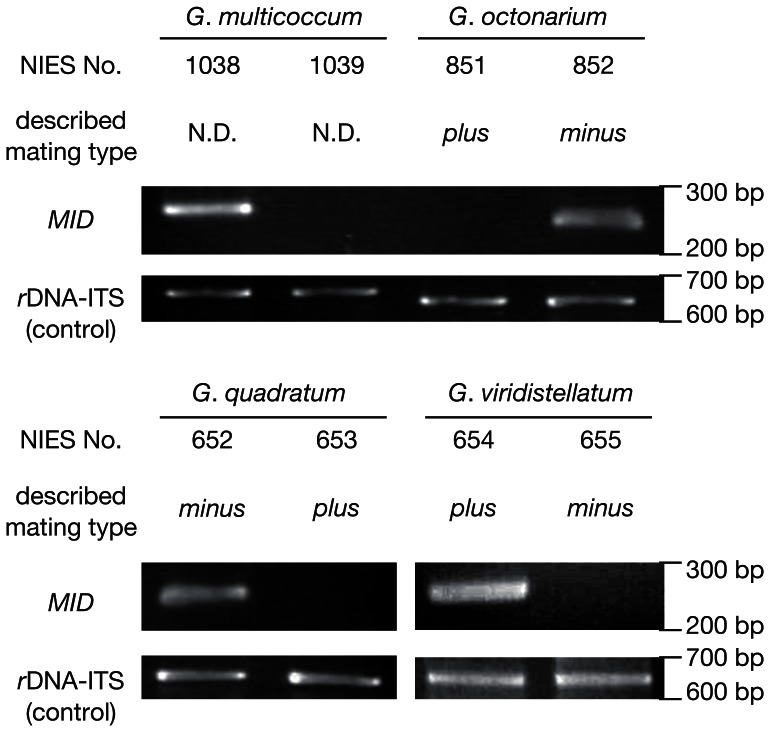
PCR assays for *MID* homolog distribution in four *Gonium* species. As a control experiment, amplification of the *r*DNA internal transcribed spacer region (ITS) is shown for each strain. Note that in *G*. *viridistellatum*, the *plus* strain is the *MID* containing strain, opposite the designation for the other *Gonium* species. N.D.: no designation.

**Figure 3:**
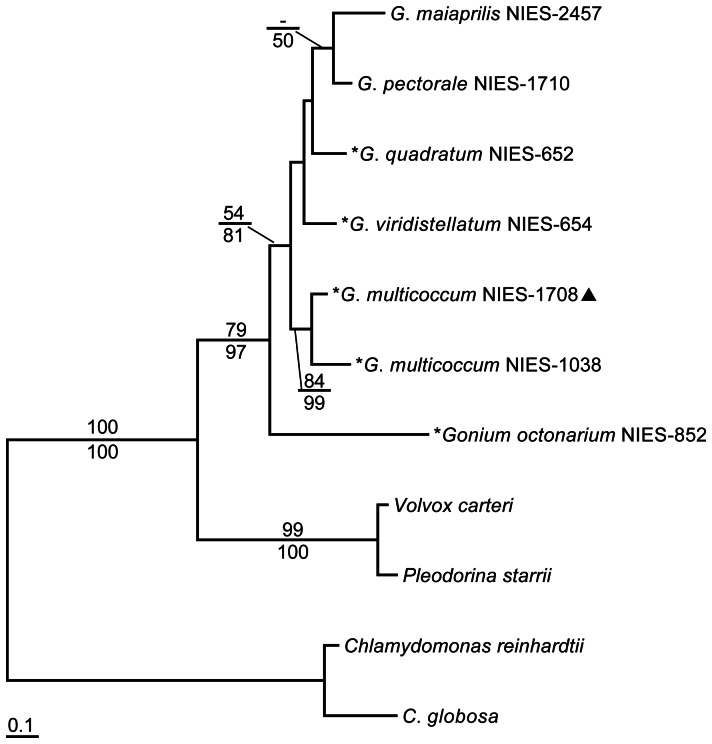
Maximum-likelihood (ML) tree (based on WAG model) of the full-length sequence of eleven MID proteins. Branch lengths are proportional to the estimated amino acid substitutions, which are indicated by the scale bar below the tree. Numbers over and below branch points indicate bootstrap values of the ML and neighbor-joining (NJ; based on the JTT model), analyses, respectively. MID homologs with asterisks (*) are reported in this study; a filled triangle indicates the homothallic strain.

**Figure 4:**
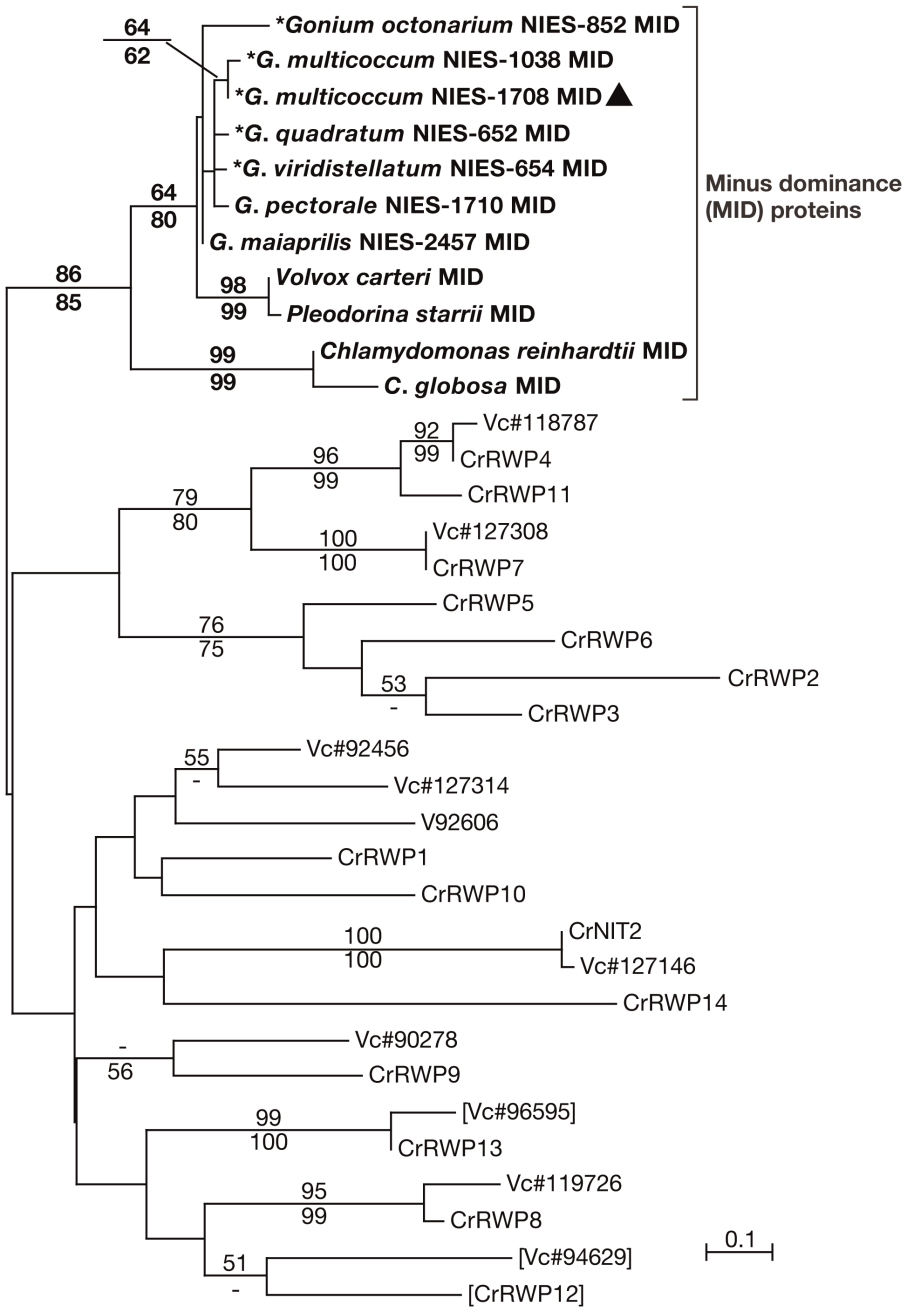
Maximum-likelihood tree (based on WAG model) of RWP-RK domains from eleven MID proteins and 25 RWP-RK domains from *C.reinhardtii* (Cr) and *V*. *carteri* (Vc) genome databases. Branch lengths are proportional to the estimated amino acid substitutions, which are indicated by the scale bar above the tree. Numbers over and below branch points indicate bootstrap values of the ML and NJ (based on the JTT model), analyses, respectively. MID homologs with asterisks (*) are reported in this study; a filled triangle indicates the homothallic strain.

As summarized in Table 1, the mating type denotations of *G*. *viridistellatum* turned out to be “inverted” in terms of *MID* distribution: only the “*plus*” strain, *G*. *viridistellatum* NIES-654, showed *MID* PCR signal (Figure 2). Phylogenetic analyses (Figures 3, 4) show that identified MID homologs are orthologous to one another among the RWP-RK domain-containing gene models recognized in *C*. *reinhardtii* and *V*. *carteri* genome databases [25], [26]. Of all the species studied here, none of the *MID* flanking regions sequenced by inverse PCR or TAIL-PCR detected an *MTD1* homolog, which is encoded closely flanking *GpMID* in *G*. *pectorale* [35].

Methods for PCR-based mating type identification of *C*. *reinhardtii* strains [36]–[38] utilized specific primers not only for *minus* but also for *plus* including *FUS1*, a *plus* specific glycoprotein-coding gene [8], [39]. Although this scheme can distinguish *plus* and *minus* reciprocally within a species, there is no *FUS1* homolog reported in the genus *Gonium* so far; *FUS1* homologs may have evolved too rapidly to be identified by degenerate primers [13]. Our genus-wide *MID* identification is not a “one-shot” identification of mutually exclusive mating types but establishes a correspondence among the different species, mating types of which have been distinguished within each morphological species.

One current and striking problem with volvocine algal strains maintained in culture collections is a decline of mating activity during long-term maintenance in vegetatively growing culture. Strains isolated several decades ago may not show mating behavior even under the sex-inducing conditions [40], [41]. Current collections of *Gonium* strains originated decades ago (Table 1). Thus, PCR-based mating type identification is *sine qua non* for many cultures in the volvocine lineage.

*MID* homologs have also been identified from male strains of two anisogamous/oogamous colonial green algae *Pleodorina starrii* and *V*. *carteri* [15], [17], indicating that isogamous *minus* and anisogamous/oogamous male share a homologous mating genotype or sex. Similarly, presence and absence of *MID* homologs may connect isogamous species with bilateral mating papilla to those that are unilateral. Unfortunately, other mating type-specific coding genes such as *FUS1* or *MTD1* in *C*. *reinhardtii* or *V*. *carteri* either do not have homologs or exhibit weak homology, unlike *MID* [17], [35], [42]. Our co-ordination framework as presence/absence of the *MID* homolog can basically be applied to other volvocine isogamous species with bilateral mating papilla such as *Pandorina* or *Yamagishiella*. Additionally, uniparental inheritance of organellar genomes changed in the course of evolution from isogamy to oogamy; in isogamous *C*. *reinhardtii*, *G*. *pectorale* and *G*. *maiaprilis*, chloroplast DNA from *plus* and mitochondrial from *minus* are inherited by the F1 progeny; in oogamous *V*. *carteri*, on the other hand, both chloroplast and mitochondrial DNA are inherited by the F1 progeny from female or *plus* [16]–[18], [43], [44]. In addition, there is very limited data on whether TMS-forming phenotypes of the organisms with unilateral papilla would be robustly associated with the *non*-*MID mating type* and hence might prove to be an uncertain indicator for sex; the mating structure of *C*. *globosa*, only a *MID mating type* of which is known, resembles that of *C*. *reinhardtii minus* [13]. Mating type/sex correspondence is the basis on which to elucidate the transitions of uniparental inheritance and mating structures.

So far, searches for *MID* homologs have been reported only in heterothallic strains. Present results clearly show that a homothallic *G*. *multicoccum* NIES-1708 strain [19] also has the *MID* homolog (Table 1 and Figure 3). When compared, non-synonymous/synonymous substitution ratios of *MID* genes from homothallic and heterothallic strains of *G*. *multicoccum* to those of the other species are below 0.2 (Table 3), indicating strong functional constrain of the genes. It seems that heterothallism in volvocine algae is ancestral; homothallism has multiple independent origins such as some strains from *G*. *multicoccum*, *G*. *pectorale* (“Russia” strain [45]), *Pl*. *japonica* [46], multiple *Eudorina* species [47], *Pandorina morum* [48], and several *Volvox* species, including most of *Volvox* sect. *Volvox* (*Euvolvox*) [48]. Gene regulatory mechanisms in homothallic strains remain unknown. In a homothallic organism, a strain established from only one vegetative cell differentiates into both gametes of sexual dimorphism, as demonstrated for the homothallic alga *Chlamydomonas monoica* [49]. The *C*. *reinhardtii iso1 mt* ^–^ mutant exhibited within a single strain an “isoagglutinating” phenotype [50] which is essentially a “partially homothallic” mode with an intact *MID* gene [51] but without any *FUS1* gene. The identification of a *G*. *multicoccum* NIES-1708 *MID* homolog suggests a *MID*-dependent mechanism is involved in the sexual developmental program of homothallic wildtype organisms. However, the homothallic strain *G*. *multicoccum* NIES-1708 does not show sexual activity in nitrogen-deficient medium now [unpublished data], possibly because mating efficiency has declined in long-term culture. Investigating expression patterns of genes homologous to mating type differentiation factors (including *MID*) requires strains newly isolated from wild samples.

**Table 3:** Non-synonymous/synonymous substitution ratio among *Gonium MID* genes.

	*G. maiaprilis*	*G. multicoccum* NIES-1038	*G. multicoccum* NIES-1708	*G. quadratum*	*G. viridistellatum*	*G. octonarium*
*G. pectorale*	0.027	0.0522	0.0441	0.0331	0.0407	0.0938
*G. maiaprilis*		0.051	0.0501	0.0476	0.0533	0.103
*G. multicoccum* NIES-1038			0.0461	0.0414	0.0406	0.0829
*G. multicoccum* NIES-1708				0.0465	0.0274	0.0789
*G. quadratum*					0.0322	0.0828
*G. viridistellatum*						0.1247

## Supporting Information

Figure S1 Multiple alignments of MID orthologs. Background colors of residues are assigned by eBioX (http://www.ebioinformatics.org/index.html).
(TIF)

Figure S2 Multiple alignments of amino-acid sequences of RWP-RK domains from volvocine algae. The prefix Cr represents genes or gene models of *Chlamydomonas reinhardtii*, while Vc *Volvox carteri* and the numbers indicate their protein IDs in the genome database. *C*. *globosa* MID is formerly identified as *C*. *incerta* MID and renamed due to taxonomic re-identification [52]. Background colors of residues are assigned by eBioX (http://www.ebioinformatics.org/index.html).
(TIF)

Table S1 The%GC and exon-intron structure in coding sequences of *Gonium MID* orthologs identified in this study.
(DOC)

Text S1 Supplementary methods.
(DOC)
