# Supplementary figures and images for: Distribution of the Sex-Determining Gene MID and Molecular Correspondence of Mating Types within the Isogamous Genus Gonium (Volvocales, Chlorophyta)
Source: PLoS One. 2013 May 16;8(5):e64385. doi: 10.1371/journal.pone.0064385 (PMC3655996; doi:10.1371/journal.pone.0064385)

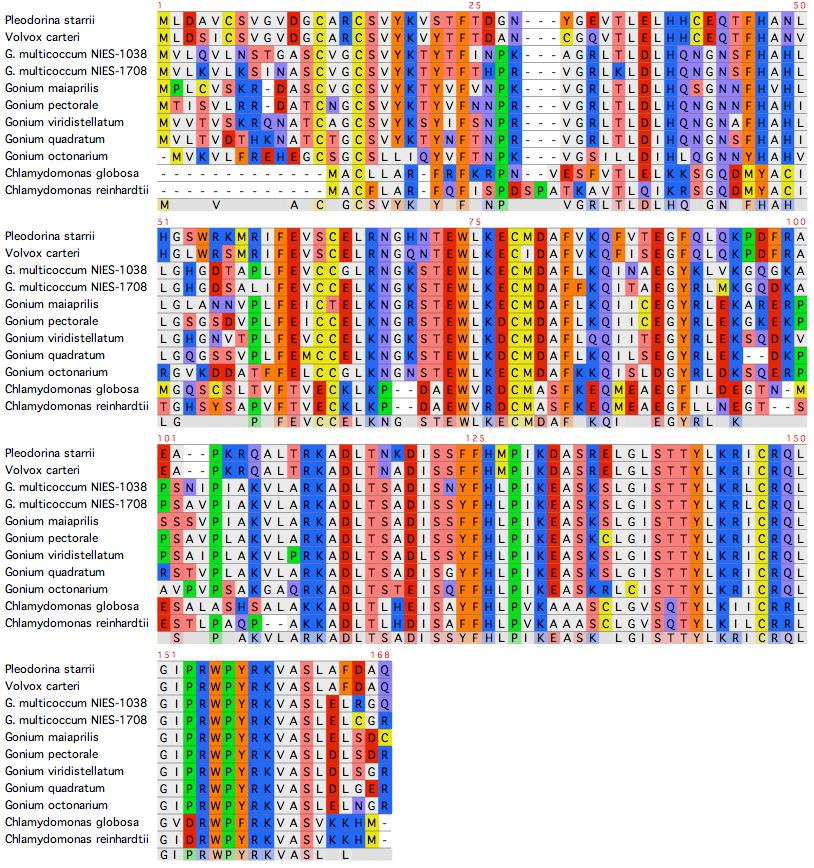

Supplement: Figure S1 — Multiple alignments of MID orthologs. Background colors of residues are assigned by eBioX (http://www.ebioinformatics.org/index.html). (TIF) [file pone.0064385.s001.tif]

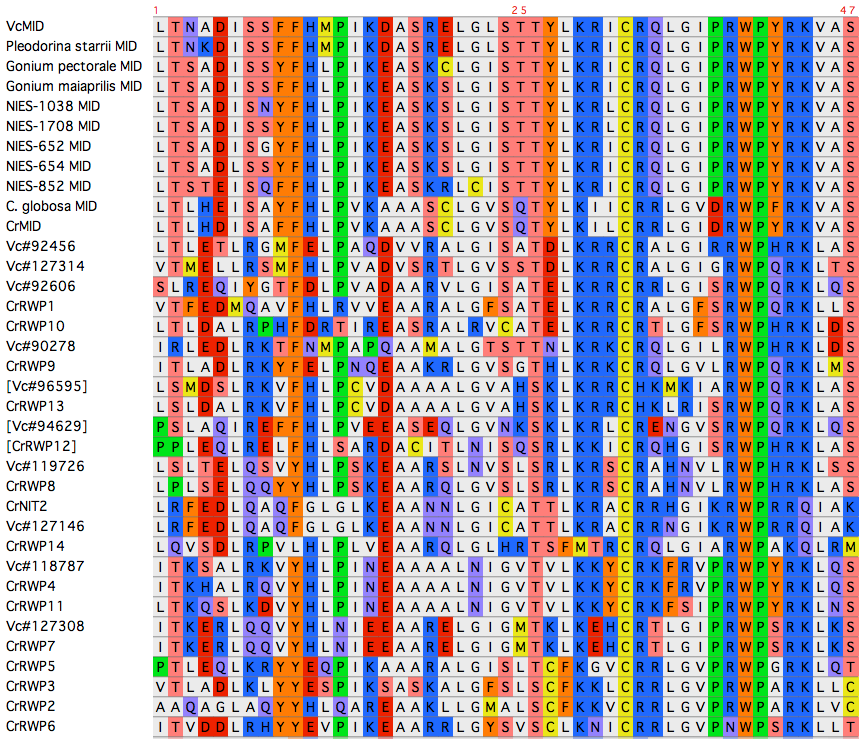

Supplement: Figure S2 — Multiple alignments of amino-acid sequences of RWP-RK domains from volvocine algae. The prefix Cr represents genes or gene models of Chlamydomonas reinhardtii, while Vc Volvox carteri and the numbers indicate their protein IDs in the genome database. C. globosa MID is formerly identified as C. incerta MID and renamed due to taxonomic re-identification [52]. Background colors of residues are assigned by eBioX (http://www.ebioinformatics.org/index.html). (TIF) [file pone.0064385.s002.tif]
