# Supplementary material for: Distribution of the Sex-Determining Gene MID and Molecular Correspondence of Mating Types within the Isogamous Genus Gonium (Volvocales, Chlorophyta)
Source: PLoS One. 2013 May 16;8(5):e64385. doi: 10.1371/journal.pone.0064385 (PMC3655996; doi:10.1371/journal.pone.0064385)
Supplement: Table S1 — The%GC and exon-intron structure in coding sequences of Gonium MID orthologs identified in this study. (DOC) [file pone.0064385.s003.doc]

Table S1

%GC and intron sizes of *Gonium* *MID* orthologs

|  | **%GC (CDS)** | exon1-CDS | **intron1** | exon2 | **intron2** | exon3 | **intron3** | exon4 | **intron4** | exon5-CDS |
| --- | --- | --- | --- | --- | --- | --- | --- | --- | --- | --- |
| *G*. *maiaprilis* | **52.7** | 82 | **55** | 100 | **52** | 98 | **57** | 185 | **54** | 30 |
| *G*. *multicoccum*  NIES-1708 (homothallic) | **50.2** | 85 | **57** | 100 | **69** | 98 | **86** | 185 | **66** | 30 |
| *G*. *multicoccum* NIES-1038 (heterothallic) | **52.4** | 85 | **59** | 100 | **58** | 98 | **61** | 185 | **75** | 30 |
| *G*. *octonarium* | **49.5** | 82 | **63** | 100 | **71** | 98 | **68** | 185 | **57** | 30 |
| *G*. *pectorale* | **52.5** | 82 | **62** | 100 | **62** | 98 | **58** | 185 | **94** | 30 |
| *G*. *quadratum* | **52.4** | 85 | **55** | 100 | **62** | 92 | **75** | 185 | **63** | 30 |
| *G*. *viridistellatum* | **51.8** | 85 | **67** | 100 | **59** | 98 | **61** | 185 | **134** | 30 |
