# Supplementary material for: Distribution of the Sex-Determining Gene MID and Molecular Correspondence of Mating Types within the Isogamous Genus Gonium (Volvocales, Chlorophyta)
Source: PLoS One. 2013 May 16;8(5):e64385. doi: 10.1371/journal.pone.0064385 (PMC3655996; doi:10.1371/journal.pone.0064385)
Supplement: Text S1 — Supplementary methods. (DOC) [file pone.0064385.s004.doc]

**Text S1**

**Supplementary methods.**

***G*. *multicoccum* NIES-1038/1039 *MID* identification.**

NIES-1038 *MID* fragment amplification was performed by nested PCR [1] with the genomic DNA sample extracted previously [2]: primers for 1st PCR were CCGMID-F1 and mt-R4; for 2nd PCR, dMT-dF3 and mt-R4; the resulting fragment was directly sequenced (all primers used in this study are summarized in Table 2). For subsequent experiments, NIES-1038 genomic DNA was isolated using the Plant Genomic Miniprep Kit (SIGMA, St. Louis, MO). The NIES-1038 *MID* flanking region was amplified by inverse PCR [3]: digests of genomic DNA with BamHI, EcoRI, HindIII or PstI were self-ligated in 2X ligation mix (Takara bio inc., Shiga, Japan); amplification by LA Taq in GC Buffer II (Takara bio inc.) as follows: 95C/5 min, followed by 35 cycles of 95C/30sec, 50C/30 sec and 72C/3 min and 72C/7 min. Resulting fragments were directly sequenced. For PCR-based *MID* distribution assay, genomic DNA samples from NIES-1038/1039 were prepared by a “colony PCR” method [4]. The specific primers used in the PCR assay for *MID* distribution were Gmul-Fwd and Gmul-Rev; ITS_a_short and ITS_b_short for the 18S *r*DNA internal transcribed spacer region [5] as control; PCR was performed as follows: 94C/2 min, followed by 35 cycles of 98C/15 sec, 55C/30 sec and 68C/30 sec using KOD FX Neo (TOYOBO, Osaka, Japan).

In the following species, PCR assay experiments were conducted using the same reaction composition and cycles, except for *MID* specific primers.

***G*. *multicoccum* NIES-1708 *MID* identification.**

Genomic DNA was isolated as previously described [6]. Nested genomic PCR was performed following *MID* fragment amplification by nested RT-PCR [6]: primers for 1st PCR were CCGMID-F1 and mt-R4; for 2nd PCR, dMT-dF3 and mt-R4; the resulting fragment was directly sequenced. Flanking regions were TA-cloned and sequenced after amplification by inverse PCR [3] (digestion, self-ligation, amplification and direct-sequencing were performed as in NIES-1038); primers used: Forward, GmMIDbF; Reverse, GmMIDaR.

***G*. *octonarium* *MID* identification.**

NIES-851/852 total RNA isolation and nested RT-PCR were performed as described previously [6] using the CapFishing full-length cDNA premix kit (Seegen, Seoul, Korea); primer pair for the 1st PCR: Forward, CCGMID-F1, Reverse, 3’-RACE primer of the kit; for the 2nd PCR: Forward, dMT-dF3 [7], Reverse, mt-R4 [6]. Genomic DNA of NIES-852 was isolated as in NIES-1038. Flanking regions were cloned and sequenced after amplification by TAIL-PCR [8] with specific primers for 5’ orientation: 1st, GoMID_Rev4; 2nd, GoMID_Rev5; 3rd, GoMID_Rev6; specific primers for 3’-orientation: 1st, GoMID_B_Fwd1; 2nd, GoMID_B_Fwd2; 3rd, GoMID_B_Fwd3. For PCR assay, specific primers Goct-Fwd and Goct-Rev were used; genomic DNA samples of NIES-851/852 were prepared by the “colony PCR” method [4].

***G*. *quadratum* *MID* identification.**

Genomic DNA samples of NIES-652 and 653 were extracted as described [9]. Genomic PCR of NIES-652 or 653 were performed using KOD FX Neo (TOYOBO) with degenerate primers: Forward, Goni-MID-F, Reverse, Goni-MID-R; cycling condition was 94C/2 min, followed by 35 cycles of 98 C/15 sec, 55C/30 sec. and 66C/30 sec; the resulting fragment from NIES-652 was cloned and sequenced. Flanking regions were amplified by TAIL-PCR [8] with specific primers for 5’-orientation: 1st, GQM-F1; 2nd, GQM-F2, 3rd, GQM-F3; specific primers for 3’-orientation: 1st, GQM-R3; 2nd, GQM-R2; 3rd: GQM-R1; the resulting fragments were directly sequenced. For the PCR assay, specific primers Gqua-Fwd and Gqua-Rev were used.

***G*. *viridistellatum* *MID* identification.**

*MID* fragment amplification was performed as in *G*. *multicoccum* NIES-1038 with NIES-654/655 genomic DNA extracted essentially as described previously [10-13]. For inverse PCR, NIES-654 genomic DNA was newly prepared with the GenElute Plant Genomic DNA Mini Kit (SIGMA). Flanking regions were cloned and sequenced after amplification by inverse PCR [3] (digestion, self-ligation, amplification and direct-sequencing as in NIES-1038); primers used: Forward, GoMID-BF; Reverse, GoMID-AR. For the PCR assay, specific primers Gvir-Fwd and Gvir-Rev were used.

**References**

1. Nozaki H, Mori T, Misumi O, Matsunaga S, Kuroiwa T (2006) Males evolved from the dominant isogametic mating type. Curr Biol 16: R1018–R1020.
2. Yamada TK, Nakada T, Miyaji K, Nozaki H (2006) Morphology and molecular phylogeny of *Gonium multicoccum* (Volvocales, Chlorophyceae) Newly Found in Japan. Jpn J Bot 81: 139–147.
3. Sambrook J, Russell DW (2001) Molecular cloning: a laboratory manual. NY Cold Spring Harbor Laboratory Press.
4. Cao M, Fu Y, Guo Y, Pan J (2009) *Chlamydomonas* (Chlorophyceae) colony PCR. Protoplasma 235:107–110
5. Coleman AW, Suarez A, Goff LJ (1994) Molecular delineation of species and syngens in Volvocacean green algae (Chlorophyta). J Phycol 30:80–90
6. Miller SM, Rüdiger S, Kirk DL (1993) *Jordan*, an active Volvox transposable element similar to higher plant transposons. Plant Cell 5:1125-1138.
7. Hamaji T, Ferris PJ, Coleman AW, Waffenschmidt S, Takahashi F, Nishii I, Nozaki H (2008) Identification of the Minus-dominance gene ortholog in the Mating-Type locus of *Gonium pectorale*. Genetics 178:283-294.
8. Liu YG, Whittier RF (1995) Thermal Asymmetric Interlaced PCR: automatable amplification and sequencing of insert end fragments from PI and YAC clones for chromosome walking. Genomics 25:674-681.
9. Newman SM, Boynton JE, Gillham NW, Randolph-Anderson BL, Johnson AM, Harris EH (1990) Transformation of chloroplast ribosomal RNA genes in *Chlamydomonas*: molecular and genetic characterization of integration events. Genetics 126:875-888
10. Nozaki H, Ito M, Sano R, Uchida H, Watanabe MM, Kuroiwa T (1995) Phylogenetic relationships within the colonial Volvocales (Chlorophyta) inferred from *rbcL* gene sequence data. J. Phycol. 31: 970-979.
11. Nozaki H, Ohta N, Yamada T, Takano H (1998) Characterization of *rbcL* group IA introns from two colonial volvocalean species (Chlorophyceae). Plant Mol Biol 37: 77-85.
12. Nozaki H, Misawa K, Kajita T, Kato M, Nohara S, Watanabe MM (2000) Origin and evolution of the colonial Volvocales (Chlorophyceae) as inferred from multiple, chloroplast gene sequences. Mol Phylog Evol 17: 256-268.
13. Nozaki H, Takahara M, Nakazawa A, Kita Y, Yamada T, Takano H, Kawano S, Kato M (2002) Evolution of *rbcL* group IA introns and intron open reading frames within the colonial Volvocales (Chlorophyceae). Mol Phylog Evol 23: 326-338.
